# Cross-linker design determines microtubule network organization by opposing motors

**DOI:** 10.1073/pnas.2206398119

**Published:** 2022-08-12

**Authors:** Gil Henkin, Wei-Xiang Chew, François Nédélec, Thomas Surrey

**Affiliations:** ^a^Centre for Genomic Regulation, Barcelona Institute of Science and Technology, 08003 Barcelona, Spain;; ^b^The Francis Crick Institute, London NW1 1AT, United Kingdom;; ^c^Sainsbury Laboratory, University of Cambridge, Cambridge CB2 1LR, United Kingdom;; ^d^Catalan Institution for Research and Advanced Studies (ICREA), Barcelona, 08010 Spain

**Keywords:** self-organization, active matter, microtubules, motor proteins

## Abstract

During cell division, cross-linking motors determine the architecture of the spindle, a dynamic microtubule network that segregates the chromosomes in eukaryotes. It is unclear how motors with opposite directionality coordinate to drive both contractile and extensile behaviors in the spindle. Particularly, the impact of different cross-linker designs on network self-organization is not understood, limiting our understanding of self-organizing structures in cells but also our ability to engineer new active materials. Here, we use experiment and theory to examine active microtubule networks driven by mixtures of motors with opposite directionality and different cross-linker design. We find that although the kinesin-14 HSET causes network contraction when dominant, it can also assist the opposing kinesin-5 KIF11 to generate extensile networks. This bifunctionality results from HSET’s asymmetric design, distinct from symmetric KIF11. These findings expand the set of rules underlying patterning of active microtubule assemblies and allow a better understanding of motor cooperation in the spindle.

## Significance

Inheritance of genetic material following chromosome duplication in eukaryotic cell division is coordinated by the spindle apparatus. The spindle is a highly interconnected network of microtubule filaments that are cross-linked by different types of molecular motors. How the different motors cooperate to organize the spindle network is not understood. Here, we show that an asymmetric cross-linker design can confer bifunctionality to a mitotic motor in the presence of other motors. The asymmetric motor supports both extensile and contractile microtubule network behaviors as observed in different parts of the spindle. These findings define rules controlling the generation of active microtubule networks and allow us to better understand how motors cooperate to organize the correct spindle architecture when a cell divides.

The mitotic spindle, which segregates the chromosomes during eukaryotic cell division, is a paradigm for self-organizing active filament networks. It is composed of dynamic microtubules, interconnected by motile cross-linkers, forming an active gel continuously driven by internal stresses while maintaining steady-state shape (1–5). The metaphase spindle can be conceptualized as a central extensile nematic network of antiparallel microtubules that gradually turns into polar microtubule networks at the spindle poles (6, 7). How cross-linking motors arrange the microtubules in such a network is not understood.

In vitro reconstitutions of self-organizing microtubule networks with purified cross-linking motors have provided insight into the basic properties of active microtubule-based networks (5, 6, 8–16). Mixtures of microtubules with a single type of cross-linking motor can form extensile, nematic networks in the presence of crowding agents which promote microtubule bundling (11, 16, 17). In the absence of crowding agents, such nematic networks can also form when microtubule densities are relatively high and cross-linking motors bundle microtubules without accumulating at microtubule ends, which happens when the ends grow too fast for motors to reach them (6). In contrast, contractile or radially polar networks form when motors can accumulate efficiently at microtubule ends, gathering them together, which is favored by conditions where motors are fast compared to microtubule growth (6, 9, 12, 13). The rules governing extensile versus contractile microtubule network formation when different cross-linking motors are present are, however, still unknown.

Moreover, natural motors have distinct designs. Kinesin-5 is a plus end–directed, symmetric motor that can cross-link two microtubules using pairs of motor domains present at either end of the tetrameric molecule (18). It slides antiparallel microtubules apart in vitro with a speed of ∼30 nm/s per motor pair (6, 19). During cell division, kinesin-5 slides spindle microtubules apart with a similar speed (8, 19–21). Kinesin-14 is a minus end–directed motor, thought to support pole-focusing activity in conjunction with dynein (22, 23). Unlike symmetric kinesin-5, kinesin-14 is an asymmetric motor that can cross-link two microtubules using one pair of motor domains and one pair of diffusible microtubule binding tail domains at opposite ends of the dimeric molecule (8, 24, 25). The speed of the motor is ∼80 nm/s (6, 10, 24, 26). The consequences of the different cross-linker designs on network formation are unknown.

Here we perform in vitro experiments with mixtures of both mitotic kinesins and microtubules nucleating in solution. We find that cross-linker design plays a particularly important role when motors with opposite directionality work together to generate an active network. Kinesin-14, mostly known as an aster-forming motor, promotes radially polar network formation when dominant but is surprisingly also able to act cooperatively with kinesin-5 to generate nematic networks. Computer simulations demonstrate that this collective behavior is a consequence of kinesin-5 being a stronger cross-linking motor than kinesin-14, which on the other hand is still an efficient microtubule bundler. Our study illustrates how the design of mitotic motors is optimized for their combined action in the spindle and provides insight into the principles of active multimotor network organization.

## Results

### The Organizational Capacities of HSET and KIF11 Diverge at High Tubulin Concentrations.

**Fig. 1.**
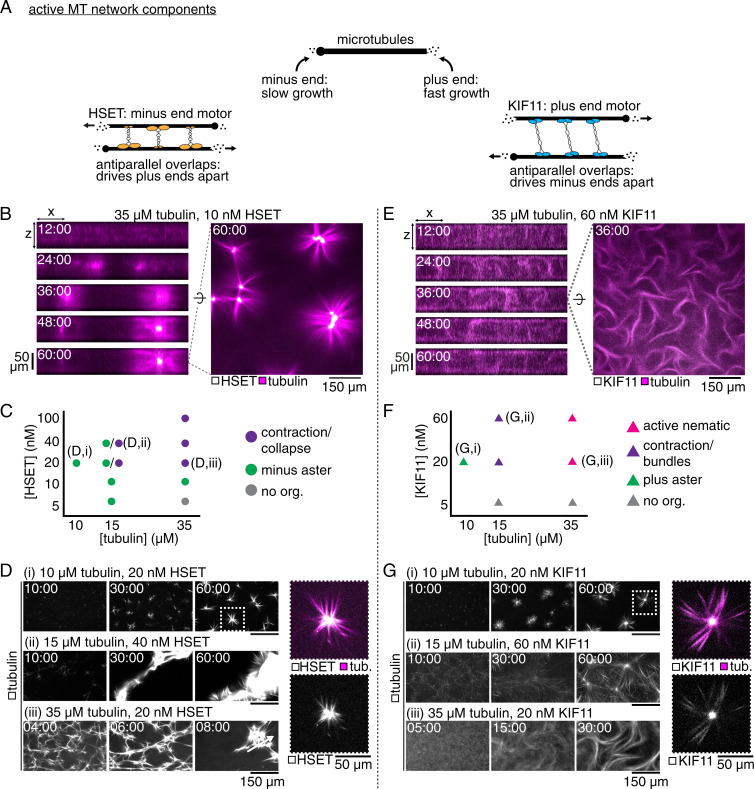
Characterizing HSET- and KIF11-driven organization of docetaxel-nucleated microtubule networks. (*A*) Schematic showing the major components of competitive motor network assembly and their properties. (*B*) Confocal imaging of the self-organization of a 35-μM tubulin network by 10 nM mCherry-HSET into separated asters. *Left*: Evolution of the network contraction into asters in an x-z plane. *Right*: Snapshot of HSET-mediated asters after 60 min of assembly in an x-y plane at the chamber’s midplane. (*C*) Phase diagram of mCherry-HSET–mediated microtubule network organization. (*D*) Time sequence of microtubule organization for three selected conditions from the phase space, showing (*i*) minus asters, (*ii*) larger-scale contraction, and (*iii*) rapid network collapse. *Right*: Enlarged image of HSET aster; mCherry signal is limited to the pole-proximal microtubule lattice. (*E*) Confocal imaging of the self-organization of a 35-μM tubulin network by 60 nM KIF11-mGFP into an active nematic network. *Left*: Evolution of the turbulent network extensile activity in an x-z plane. *Right*: Snapshot of KIF11-mediated extension after 36 min of assembly in an x-y plane at the chamber’s midplane. (*F*) Phase diagram of KIF11-mGFP–mediated microtubule network organization. (*G*) Time sequence of microtubule organization for three selected conditions from the phase space, showing (*i*) plus asters, (*ii*) loosely contractile bundles, and (*iii*) active nematic activity. *Right*: Enlarged image of KIF11 aster; mGFP signal is highly enriched at the pole but still appears on distal microtubule lattice. See also Movie S1.

Recently, we systematically investigated the organizational phase space of microtubule self-organization driven by the symmetric cross-linker kinesin-5 (6). The capabilities of an asymmetric cross-linker, such as kinesin-14, to make diverse networks have not yet been studied in a similarly systematic fashion. Using a microscopy-based motor/microtubule self-organization assay, we first investigated what network morphologies each type of cross-linker could produce on their own (Fig. 1*A*), before studying how symmetric and asymmetric motile cross-linkers with opposite directionality work together. To promote efficient microtubule nucleation from soluble tubulin, we added low concentrations of the microtubule-stabilizing drug docetaxel, whose relatively high solubility compared to other taxanes was found to be advantageous (*Methods*) (27). Under these conditions, microtubules polymerize until the tubulin subunits have been consumed and the microtubules are then stable. Microtubule growth is intrinsically asymmetric, with plus ends growing faster than minus ends.

Purified minus end–directed human kinesin-14 HSET generated contractile microtubule networks as observed previously (6, 8, 10). At a tubulin concentration of 35 μM and an HSET concentration (all motor concentrations refer to monomers) of 10 nM, well-focused, separated asters formed (Fig. 1*B*, *Right*). Asters became centered between the top and bottom of the experimental chamber and HSET accumulated in the middle of the asters, indicating that the minus ends were gathered (Fig. 1*B*, *Left*). Asters that came into contact fused (Movie S1*A*). We explored the phase space of network organization by HSET (Fig. 1*C*). Well-defined aster formation was favored at relatively low tubulin concentrations (10 µM) and intermediate HSET concentrations (20 nM) (Fig. 1*D*, *i*). At increased concentrations of tubulin (15 μM) and HSET (40 nM), when networks are expected to be more percolated, they could contract as large entities (Fig. 1*D*, *ii*), as seen previously in contractile motor-driven microtubule networks (6, 8, 13, 28) and cross-linked actin-myosin networks (29). Contraction was fastest at high tubulin concentrations (35 µM), when microtubules nucleated and elongated at faster rates (Fig. 1*D*, *iii*, and *SI Appendix*, Fig. 1). The overall contractile nature of HSET/microtubule networks is due to the relatively slow growth speed of microtubule minus ends compared to the HSET motor speed, allowing HSET to gather microtubule minus ends over a wide range of conditions (6).

In contrast, purified plus end–directed human kinesin-5 KIF11 generated nematic networks of extensile bundles with evenly distributed KIF11 when the tubulin concentration was high (35 µM) (Fig. 1*E*, *Right* and Movie S1*B*) (6). The microtubule plus-end growth speed is high at early times under these conditions, which prevents KIF11 from gathering plus ends into contractile structures. Instead, KIF11 drives persistent sliding of antiparallel microtubules, which in the dense microtubule suspension leads to repeated buckling, merging, and sliding of bundles in three dimensions (Fig. 1*E*, *Left*) (6, 11, 14–16, 19). We varied tubulin and KIF11 concentrations (Fig. 1*F*). At lower tubulin concentrations (10 µM), at which microtubule plus ends grow more slowly, KIF11 formed contractile networks and individual asters, as it could now accumulate at microtubule plus ends and bring plus ends together (Fig. 1*G*, *i*) (6, 13, 19). Bundling and bundle extension was favored at higher tubulin concentrations (15 to 35 µM) (Fig. 1*G*, *ii* and *iii*). These results agree with previous work where microtubules were nucleated differently (6) and establish the organizational phase space for networks of docetaxel-nucleated microtubules organized by a single type of motile cross-linker, either HSET or KIF11.

The organizational capacity of the two motors at low tubulin concentrations is similar, but diverges at higher tubulin concentrations. This can be understood as a consequence of the opposite directionalities of the motors and the different growth speeds at the two microtubule ends. When overall microtubule growth is faster, it is more difficult for KIF11 to accumulate at the faster-growing plus ends, whereas HSET can still accumulate at the slower-growing minus ends (6, 30).

### KIF11 Antagonizes Microtubule Aster Formation by HSET.

Next, we set out to determine the network patterns formed in the presence of both motors.

**Fig. 2.**
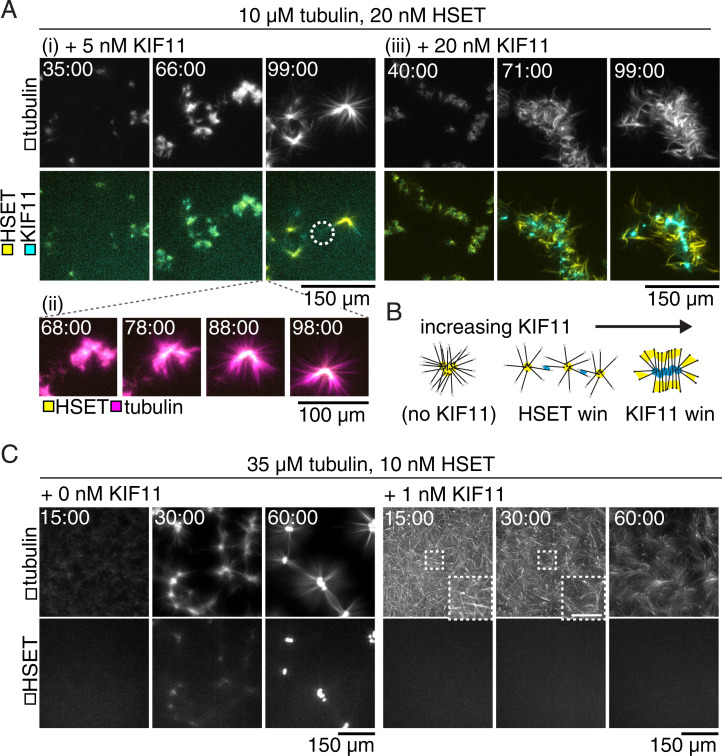
HSET and KIF11 compete to organize microtubule networks. Time courses show maximum projections of confocal images taken around the chamber midplane. (*A*) mCherry-HSET and KIF11-mGFP (*Bottom* panels) compete to contract low-density microtubule networks (*Top* panels). (*i*) KIF11-mGFP (5 nM) frustrates the assembly of asters in 10-μM tubulin networks (compare to Fig. 1*C*) and eventually appears as a diffuse signal decorating the microtubules between mCherry-HSET contractile centers (circle). (*ii*) Localization of HSET within the microtubule structure coincides with the radial appearance of microtubule ends. (*iii*) KIF11-mGFP (20 nM) dominates assembly of locally contractile structures and localizes to the structure centers. (*B*) Schematic of competitive assembly of contractile microtubule networks by KIF11-mGFP and mCherry-HSET. (*C*, *Left*) mCherry-HSET (10 nM; *Bottom* panels) contracts microtubules (*Top* panels) assembled from 35 μM tubulin into asters with well-defined centers (as in Fig. 1*B*). *Right*: The addition of 1 nM KIF11-mGFP prevents HSET contraction. mCherry-HSET signal remains diffuse within the microtubule network. Microtubule bundles (*Insets*) increase in curvature, indicating strain within the network. (Scale bar, 50 µm.) See also Movie S2.

First, we studied low tubulin concentration networks under conditions where each motor individually would drive contractile network behavior or generate asters, however with opposite polarity. When KIF11 was present at a fourfold lower concentration than HSET, KIF11 delayed HSET-driven aster formation (Fig. 2*A*, *i* and Movie S2*A*). Initially, microtubules appeared to be heavily cross-linked into dense clusters without obvious orientation or polarity. Over time, these clusters reorganized into asters with central HSET accumulation, revealing that when in excess HSET can outcompete KIF11 under aster-promoting conditions (Fig. 2*A*, *ii*). Network organization took more time with both motors being present than with each motor individually, suggestive of a sustained competition for dominance. The presence of KIF11 between HSET-enriched centers prevented these polar structures from fusing, despite persistent contact (Fig. 2*A*, *i*).

At equimolar monomer concentrations, the two motors again formed densely cross-linked microtubule clusters which coalesced as they came into contact. The motors subsequently self-sorted (Fig. 2*A*, *iii* and Movie S2*B*). KIF11 resided at the center of the assembled structures, indicating that it had successfully gathered the plus ends and dominated the contractile network behavior. HSET was relegated to the periphery where it bundled microtubules, but did not appear to efficiently gather minus ends (Fig. 2*A*, *iii*). Thus, in the low tubulin, contractile regime, the motor ratio determines which motor wins the competition (Fig. 2*B*), with KIF11 being the more efficient competitor.

The dominance of KIF11 in driving network behavior was even more striking at high tubulin concentrations. At a concentration of HSET that causes the formation of well-defined, large asters in the absence of KIF11 (Fig. 2*C*, *Left*), the addition of a 10-fold lower concentration of KIF11 completely inhibited aster formation or any local accumulation of HSET (Fig. 2*C*, *Right*). Instead, a cross-linked mesh of curved microtubule bundles formed, characteristic of extensile network behavior. These results show that in dense networks with higher microtubule growth rates, in which HSET forms asters only within a narrow range of concentrations (Fig. 1*C*), KIF11 is a particularly efficient competitor for HSET.

### Tubulin Concentration Is a Control Parameter Influencing Nematic versus Radially Polar Organization in Mixed Motor Networks.

**Fig. 3.**
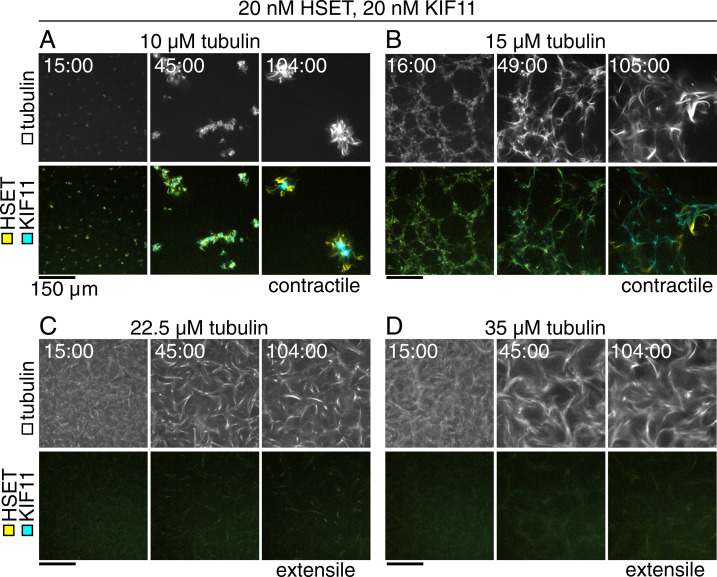
Increasing tubulin concentration drives transition from contractile to extensile gels in competitive motor-driven networks. Time course of confocal images taken in the flow chamber midplane of microtubules (*Top* panels) organized by 20 nM mCherry-HSET and 20 nM KIF11-mGFP (*Bottom* panels). (*A*) As in Fig. 2*A*, microtubules polymerized from 10 μM tubulin are contracted and form compact, locally connected structures. KIF11 is localized to the structure center, and HSET is excluded to the periphery. (*B*) Tubulin networks (15 μM) form contractile webs with long-range interactions. KIF11 signal is internal to the contracting web, and HSET localizes to bundles extruding from the network. (*C*) Tubulin (22.5 μM) assembles into microtubules that form extensile bundles. Motors are well distributed in the bundles, but HSET localization at bundle tips can be seen emerging from the extensile network. (*D*) Tubulin (35 μM) assembles into microtubules that form extensile bundles. Differential localization of the motors within the same time frame is less clear. (Scale bars, 150 μm.)

We saw a marked difference in mixed motor network organization outcomes between low and high tubulin concentrations. To investigate the effect of microtubule growth speed and density on network formation in the presence of the two motors, we allowed networks to organize at increasing tubulin concentrations while keeping the HSET and KIF11 concentration constant, at levels where KIF11 is dominant (Fig. 3). At low tubulin concentrations, contractile networks formed, and KIF11 eventually localized to astral centers (Fig. 3*A*, same conditions as in Fig. 2*A*, *iii*). Increasing the tubulin concentration led to more long-range microtubule bundling and less local contraction (Fig. 3*B* and *C*). Motor self-sorting to different bundles of the network was increasingly hindered with increasing tubulin concentrations. At the highest tubulin concentration tested, the network was mostly nematic, displaying extending bundles and evenly distributed motors (Fig. 3*D*).

In conclusion, increasing microtubule density and growth speed by increasing the tubulin concentration changed the nature of the “mixed motor” network from contractile to nematic, similar to the trend in “KIF11 only” networks (Fig. 1*F*). HSET could accumulate locally in bundles but could not efficiently generate focused minus ends. HSET delayed, but did not prevent, KIF11-driven network organization, indicating that HSET plays an antagonistic role when KIF11 dominates.

### HSET Supports KIF11 in Driving Nematic Network Formation at Low KIF11 Concentrations, Revealing Multifunctionality.

**Fig. 4.**
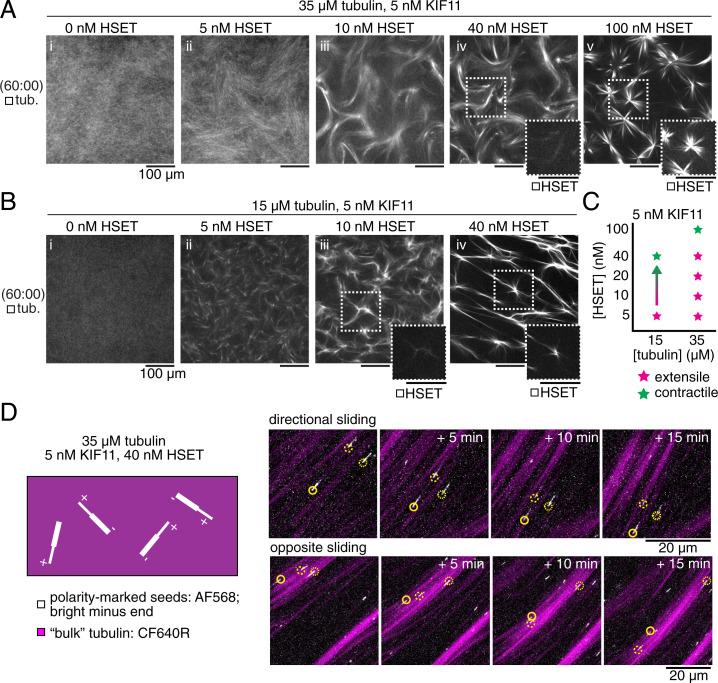
HSET facilitates extensile behavior in mixed motor networks consistent with plus-end directionality. (*A* and *B*) Snapshots show midplane confocal slices of the experimental chamber after 60 min of self-assembly. (*A*) Addition of (*i*) 5 nM to (*iv*) 40 nM mCherry-HSET drives extensile behavior of 35-μM tubulin networks in the presence of 5 nM KIF11-mGFP, which is insufficient to drive visible organization on its own. (*v*) HSET (100 nM) drives contractile organization. HSET localizes to extensile bundles and contractile centers (*Insets*). (Scale bars, 100 µm.) (*B*) Addition of (*ii*) 5 nM HSET drives extension of 15 μM tubulin in the presence of 5 nM KIF11, whereas 15-μM tubulin networks with either motor alone only show contractile behavior (see Fig. 1 *C* and *F*) or, as in the case of (*i*) 5 nM KIF11, no organization. Addition of (*iii*) 10 to (*iv*) 40 nM HSET,shows a tendency toward contraction of the network, where mCherry-HSET localizes to contractile centers (*Insets*). (Scale bars, 100 µm.) (*C*) Results summarized in a phase space plot. (*D*) Time courses show cropped maximum projections of confocal images taken in thin (20-μm) chambers. A small number of seeds with bright minus ends (AlexaFluor568) demonstrate individual microtubule activity in a background of tubulin with a spectrally separate fluorescent dye (CF640R). Arrows indicate the direction of the microtubule plus end. Microtubules in networks assembled from 35 μM tubulin, 5 nM KIF11-mGFP, and 40 nM mBFP-HSET generally move toward their minus ends (*Top* panels) and could be seen moving in opposite directions within aligned bundles (*Bottom* panels). See also Movies S3–S5.

Next, we investigated more closely the high-tubulin concentration regime, in which the independent organizational capacities of the two motors diverge, with HSET forming contractile and KIF11 extensile networks (Fig. 1). Keeping the tubulin concentration constant, we added increasing concentrations of HSET to KIF11 kept at a concentration that was too low to organize a macroscopic network state (Fig. 4*A*, *i* and Movie S3, *Top Left*). We found that the addition of an equimolar amount of HSET resulted in weak nematic network formation with some extensile activity (Fig. 4*A*, *ii* and Movie S3, *Top Right*). Increasing the HSET concentration twofold and eightfold produced increasingly active nematic networks with long, dense microtubule bundles (Fig. 4*A*, *iii* and *iv* and Movie S3, *Bottom Left*). This is remarkable because HSET on its own is unable to promote nematic network formation in these experimental conditions (Fig. 1*C*). HSET was distributed throughout the bundles when included at an eightfold excess of HSET over KIF11 (Fig. 4*A*, *iv*). Increasing the HSET concentration further to a 20-fold excess over KIF11 finally caused the network to become contractile; initially, microtubules assembled into bundles that eventually reformed into aster-like structures (Fig. 4*A*, *v* and Movie S3, *Bottom Right*).

HSET can support KIF11 to drive the formation of extensile bundles also at lower tubulin concentrations (Fig. 4*B* and Movie S5) which otherwise promote contractile activity with either motor alone (Fig. 1*C* and *F*). The range of HSET concentrations that assist extensile bundle formation driven by KIF11 is, however, decreased compared to high-tubulin concentration networks (Fig. 4*B*, *i*–*iii*). This is a consequence of contractile behavior being recovered more easily in these sparser networks with fewer and slower-growing microtubules, and aster-like structures are recovered already at an eightfold excess of HSET over KIF11 (Fig. 4*B*, *iv* and *C*). Motors may accumulate more efficiently at microtubule ends, which then promotes network contraction and aster formation.

The asymmetric cross-linker HSET may assist the symmetric cross-linker KIF11 to drive nematic network formation of extensile bundles by providing additional microtubule cross-links despite losing the competition for directional sliding. This is likely due to the weaker processivity of the HSET motor and its diffusive microtubule binding tail domain that prevents strong force production, which would mean that KIF11 drives microtubule sliding in these mixed motor nematic networks (24, 31, 32). To test this hypothesis, we added polarity-marked seeds to high-tubulin concentration networks with an eightfold excess of HSET over KIF11 and found that the majority of seeds were indeed moving with their minus end leading within the bundles (Fig. 4*D*, *SI Appendix*, Fig. 2, and Movie S5), indicating KIF11-driven microtubule sliding. Some seeds displayed saltatory motility, indicative of a “tug of war” in some parts of the network (8, 33), although the overall macroscopic dynamics were dominated by KIF11.

These experiments demonstrate that the asymmetric design of HSET, with its motor domains positioned opposite the diffusible microtubule binding tail domains, turns this motor into a multifunctional cross-linker that can either assist KIF11 in nematic network formation or promote contractile network and aster formation depending on the relative motor concentrations.

### Computer Simulations with Symmetric and Asymmetric Cross-linkers Recapitulate Experimental Network Organization.

**Fig. 5.**
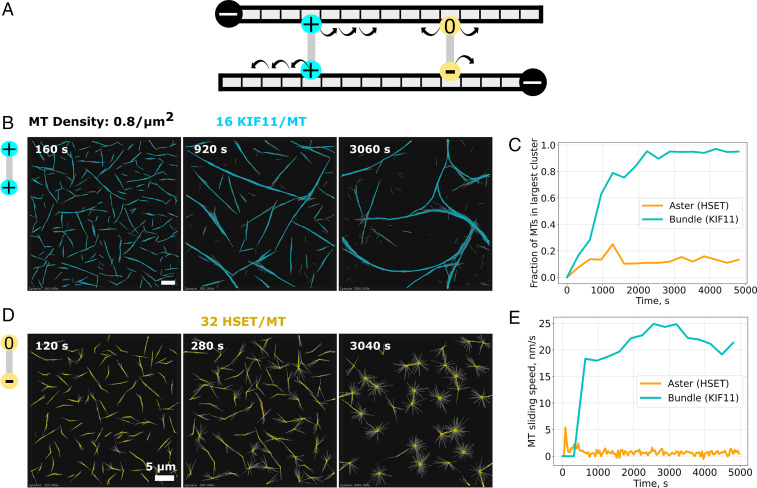
Simulated KIF11 and HSET-driven microtubule organization. (*A*) Model schematic of KIF11 (cyan) and HSET (yellow) cross-linkers. KIF11 is a symmetrical cross-linker composed of two plus end–directed motor units connected by a spring-like link. HSET is an asymmetrical cross-linker made of one minus end–directed motor unit and one diffusive unit. (*B*) Time course of simulated KIF11-driven extensile nematic bundle formation in a thin three-dimensional box (dimension: 60 µm × 60 µm × 0.2 µm). The 2,880 microtubules are colored in gray, and the 46,080 KIF11 in cyan. The ratio of KIF11 molecules per microtubule (MT) is 16. (*C*) Time course of the size of the largest bundle (cyan line) and aster (orange line) from the same simulation. Sizes were normalized by the total number of microtubules. (*D*) Time course of simulated HSET-driven aster formation. The simulation space is 40 µm × 40 µm × 0.2 µm with 1,280 microtubules and 40,960 HSET. The ratio of HSET molecules per microtubule (MT) is 32. Scale bars, 5 µm. Model parameters are given in *SI Appendix*, Table S1. (*E*) Time course of the average displacement speed of microtubules in KIF11-driven (cyan line) and HSET-driven (orange line) networks. Total simulated time is 5,000 s. See also Movie S6.

To pursue our investigation of these mixed motor microtubule networks, we turned to computer simulations. Microtubules were modeled in a thin three-dimensional space as diffusing bendable lines repelling each other via soft-core interactions (34, 35). They grew from a fixed number of nucleators by plus-end elongation for a defined period of time and then kept a fixed length (*SI Appendix*, Fig. 3*A*), mimicking the behavior of the docetaxel-stabilized microtubules in the experiments. KIF11 was modeled as a symmetric complex consisting of two plus end–directed and processive motor units mechanically connected by a Hookean spring with nonzero resting length (Fig. 5*A*) (34). KIF11 units unbound immediately upon reaching microtubule plus ends (*Methods*). In contrast to previous microtubule network simulations, we explicitly considered here the asymmetric nature of HSET. It was modeled as an asymmetric cross-linker with one minus end–directed, nonprocessive motor unit and one diffusive unit (the HSET tail domain) (Fig. 5*A*). The dwell time of the diffusive unit was 500 times larger than that of the motor unit, to reflect the experimentally measured properties of this motor (see *SI Appendix*, Table S1 for model details) (24). Both HSET units dwell at microtubule ends, not detaching immediately (*Methods*).

We first examined networks driven solely by HSET and KIF11. In the absence of any motor, microtubules remained randomly oriented (*SI Appendix*, Fig. 3*B*). At a microtubule density of 0.8 microtubules/µm^2^, KIF11 organized microtubules into bundles that continuously extended, broke apart, and reformed again (Fig. 5*B* and Movie S6*A*), similar to experimentally observed active nematic networks driven by KIF11 at high tubulin concentrations (Fig. 1*E*). To characterize the network, we extracted key quantities from the simulations: the number of cross-linkers in each configuration, the number of microtubules in a cluster, and the speed at which microtubule moved (*Methods*). In KIF11-driven networks, the average microtubule sliding speed increased with the number of microtubules becoming incorporated in the mixed polarity bundles (Fig. 5*C* and *E*, cyan). In contrast, HSET bundled and organized microtubules into asters with focused minus ends (Fig. 5*D* and Movie S6*B*) leading to immobile microtubules (Fig. 5*C* and *E*, orange), similar to experimentally observed asters generated by HSET (Fig. 1*B*). This shows that an asymmetric cross-linker with one diffusive and one motor unit, even lacking processivity, can form asters, without requiring larger motor oligomer formation (10). Compared to simulations with a hypothetical symmetric minus end–directed motor, made of two mildly processive motor units, the modeled HSET cross-linker drove assembly of asters with less focused centers and caused microtubules to be more bundled close to the aster center (*SI Appendix*, Fig. 4, *Left* and *Middle*), similar to experimentally observed HSET asters (Fig. 1*D*, *i*, *Inset*), further validating the HSET model.

### HSET Activity Synergizes with or Antagonizes KIF11 Depending on Relative Abundance.

**Fig. 6.**
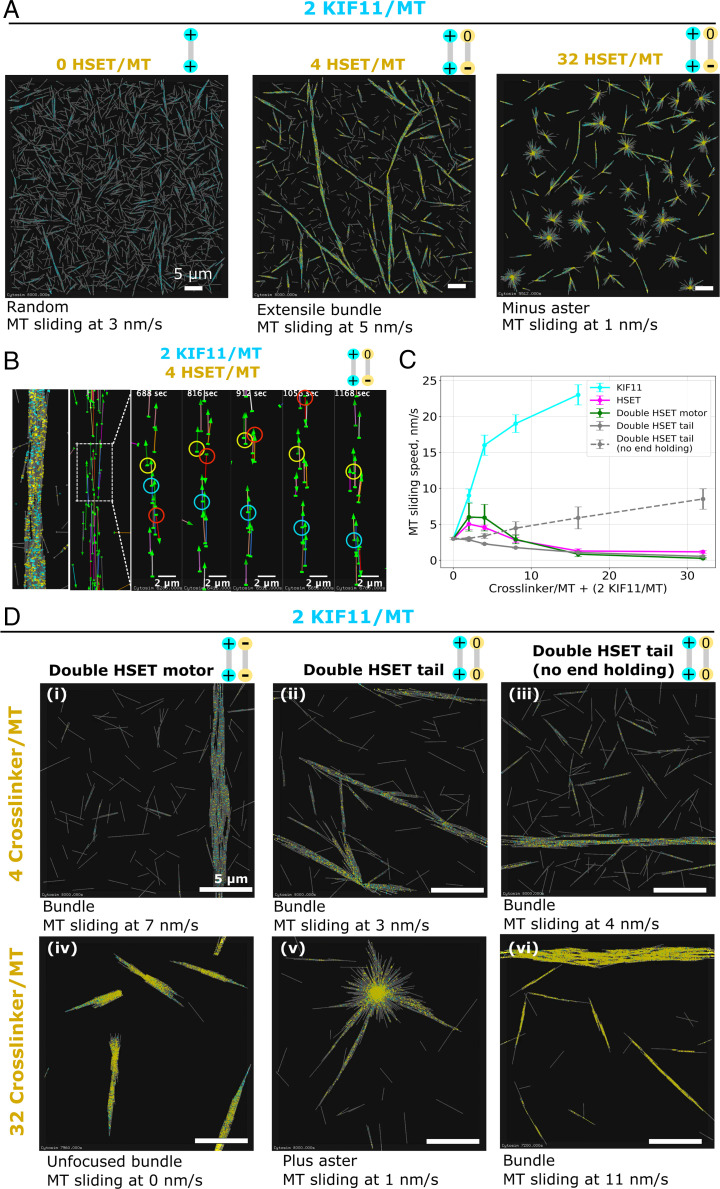
Simulated microtubule networks organized by mixed motors. (*A*) Mixing a small amount of KIF11 with an increasing amount of HSET. *Left*: Random microtubule organization in the presence of a small amount of KIF11 (cyan) only. *Middle*: Extensile bundles form when HSET (yellow) is added to KIF11 at a 2:1 ratio. *Right*: Minus asters form when HSET and KIF11 are mixed at a 16:1 ratio. The simulation space is 60 µm × 60 µm × 0.2 µm with 2,880 microtubules and 5,760 KIF11, yielding a ratio of 2 KIF11 per microtubule (MT). Total simulated time is 8,000 s. See also Movie S7. (*B*) Visualization of the trajectories of selected individual microtubules for the mixed motor condition with two HSET per KIF11. The majority of microtubules, depicted as arrows (arrow heads point in plus-end direction), move unidirectionally with the minus end leading, a sign of KIF11-driven sliding (red and cyan circles). Back-and-forth motion occurs occasionally (yellow circles). See also Movie S8. (*C*) Average microtubule sliding speed as a function of cross-linker number for various types of cross-linker (as indicated) mixed with a fixed amount of KIF11 (two KIF11 per microtubule (MT)). Each point represents a speed averaged from five sample simulations run in a box of size 20 µm × 20 µm× 0.2 µm. (*D*) Microtubule networks generated by mixtures of HSET cross-linker variants (yellow) and a small amount of KIF11 (cyan). HSET cross-linker variants are made of either two HSET motors (*i* and *iv*), two HSET tails (*ii* and *v*), or two modified HSET tails that immediately unbind when reaching a microtubule end (*iii* and *vi*). *Top Row*: 4 modified HSET cross-linkers per microtubule (MT). *Bottom Row*: 32 modified HSET cross-linkers per microtubule. Simulation space is 20 µm × 20 µm × 0.2 µm with 320 microtubules. Total simulated time is 8,000 s. All simulations contain the same density: 0.8 microtubule/µm^2^.

To determine why and under which conditions HSET either antagonizes or cooperates with KIF11, we simulated microtubules with few KIF11 cross-linkers, insufficient to organize microtubules (Fig. 6*A*, *Left* and Movie S7*A*). Adding a low amount of HSET cross-linkers promoted bundle formation (Fig. 6*A*, *Middle* and Movie S7*B*). Visualizing only some of the microtubules in the simulated bundles revealed mostly KIF11-driven microtubule sliding and occasionally saltatory movement, resembling experimental observations (Fig. 6*B* and Movie S8) (8, 33). These simulations demonstrate that, as in the experiments (Fig. 4), despite its opposite directionality, low amounts of HSET assist KIF11-driven extensile bundle formation. That is because HSET can contribute efficiently to bundling, even though it can only produce weak microtubule sliding forces (*SI Appendix*, Fig. 5) as a consequence of the diffusive binding mode of its tail (31). Nevertheless, the average microtubule sliding speed in these mixed motor bundles is slower than in KIF11-only bundles, indicating that HSET cross-linking does generate an opposing frictional force slowing down KIF11-driven sliding (Fig. 6*C*). Further increasing the amount of HSET reduces the microtubule sliding speed (Fig. 6*C*) and at even higher amounts leads to HSET-driven aster formation, with microtubules becoming immobilized by anchoring of their minus ends in the aster center (Fig. 6*A*, *Right* and *C* and Movie S7*C*), again similar to the experiments (Fig. 4*A*). Thus, while HSET assists KIF11 when present in low amounts, at high amounts HSET antagonizes and outcompetes KIF11.

### The Asymmetric Design of HSET Explains Its Multifunctional Nature.

To understand which properties of HSET make it able to either synergize with or antagonize KIF11, we simulated variations of the HSET model. Low amounts of a hypothetical plus end–directed HSET variant assisted low amounts of KIF11 to generate extensile bundles (*SI Appendix*, Fig. 6*A*, *i*), similarly to normal HSET, but leading to an increased speed of plus end–directed sliding. Further increasing the concentration of this “plus HSET” caused the formation of inverted asters with focused plus poles (*SI Appendix*, Fig. 6*A*, *ii*). These results demonstrate that the directionality of the poorly processive HSET motor does not determine its ability to assist KIF11 in extensile bundle formation, but determines aster polarity when HSET is dominant. Another hypothetical HSET, now with normal minus directionality but with a nondiffusive microtubule binding unit, also assisted KIF11-mediated extensile bundle formation (*SI Appendix*, Fig. 6*B*, *i*). This nondiffusive HSET variant, however, slowed down KIF11-driven microtubule sliding more than normal HSET (*SI Appendix*, Fig. 6*B*, *iii*). Microtubule sliding was faster when the unbinding rate of the hypothetical nondiffusive HSET tail was increased together with the number of nondiffusive HSETs to maintain a similar number of cross-links (*SI Appendix*, Fig. 6*B*, *ii* and *iii*). This shows that the diffusive nature of the HSET tail allows it to be bound to the microtubule for some time without presenting too much resistance to KIF11-driven microtubule sliding.

Next, we combined the different HSET subunits into symmetric constructs. Symmetric cross-linkers consisting of two identical HSET motor units (“double-motor HSET”) were equally efficient as the normal HSET in helping KIF11 to organize microtubules into bundles (Fig. 6*D*, *i*). The bundles extended, driven by KIF11, as long as a low amount of double-motor HSET was present (Fig. 6*C*). Higher amounts of double-motor HSET promoted stronger bundle formation but failed to generate asters (Fig. 6*D*, *iv*). Instead, microtubules were polarity sorted into bundles with double-motor HSET accumulating weakly at the microtubule ends. This demonstrates that the presence of the long-dwelling HSET tail is important for HSET’s ability to form asters. The long dwell time of the HSET tail allows its nonprocessive motor to quickly rebind to a microtubule when tethered in a bundle, effectively increasing its processivity (26) and hence its ability to efficiently gather microtubule minus ends.

Adding low amounts of another symmetric cross-linker variant, now consisting of two diffusive HSET units (“double-tail HSET”), also helped KIF11 to form microtubule bundles (Fig. 6*D*, *ii*). Bundling was more efficient than with normal HSET, given the long dwell time of the two diffusive units. However, KIF11-driven bundle extension was slower as a consequence of the increased friction produced by the diffusive units. Surprisingly, at higher amounts of double-tail HSET, a qualitatively different network formed: KIF11-driven asters with plus-end poles (Fig. 6*D*, *v*). In these asters, long-lived double-tail HSET cross-links could keep plus ends of microtubules together after they were slid apart by KIF11 (*SI Appendix*, Fig. 6, *Right*). KIF11-driven plus-end aster stabilization by double-tail HSET required the ability of the diffusive unit to hold on to microtubule ends. When the diffusive units in the model were allowed to unbind from microtubule ends, KIF11-driven extensile bundles formed instead of plus-end asters (Fig. 6*D*, *iii* and *vi*).

Neither of these simulated symmetric HSET variants replicated the full range of extensile and contractile microtubule networks driven by HSET in the presence of KIF11. Therefore, the asymmetric design of HSET is key to its microtubule organizing properties. First, the diffusive tail domain allows sustained tethering to the microtubule lattice and, coupled with a nonprocessive motor domain with rapid binding/unbinding rates, this facilitates bundling and presents low effective friction to KIF11-driven sliding. Second, these same features can also promote gathering of minus ends in local contractile structures due to effective minus end–directed movement of the microtubule-tethered HSET motors. Therefore, the design of the asymmetric cross-linker HSET combines properties that favor context-dependent microtubule bundling or aster formation. This unique set of properties makes HSET multifunctional, supporting either plus motor–driven nematic network formation when present in lower numbers or minus-end aster formation when dominant.

## Discussion

We observed both contractile and extensile network states in active microtubule networks generated by motors with opposite directionality, depending on system composition. Due to the asymmetric design of the minus motor HSET and the symmetric design of the plus motor KIF11, the two motors behaved differently. They do compete, but especially in the higher microtubule density regime, HSET can also assist KIF11 in nematic network formation, effectively creating a cross-linker with two functionalities depending on context.

HSET’s asymmetric design, characterized by a nonprocessive, minus end–directed motor that is diffusively anchored to a second microtubule for relatively long times, allows efficient aster formation in the absence of other motors, although it does not focus microtubule minus ends into very tight poles. Against an opposing motile cross-linker like KIF11 with two processive motors, HSET’s weak force-producing capacity as a consequence of its tail’s diffusive binding (31) requires a large excess over KIF11 to be able to compete efficiently and to impose minus aster formation. At similar concentrations of the two motors, new behavior emerges: HSET contributes to microtubule bundling and subsequently loses the competition for microtubule sliding, now effectively assisting KIF11-driven nematic network formation, slowing down microtubule sliding only mildly.

We previously demonstrated how the characteristically asymmetric properties of microtubule growth affect the generation of active networks by a single type of motor. Fast growth of microtubule plus ends favors nematic network formation by plus end–directed cross-linkers, whereas slow (or no) minus end growth favors the formation of asters with a minus pole by minus end–directed cross-linkers (6). This raised the interesting question: Going beyond previous studies where only aster regimes were accessible (8, 12), which network types dominate when opposite directionality motors are mixed? We showed here that the emergent phenomena in mixed motor networks can be explained by the cross-linking structures of the motors, and not only by the simple rules that determine organization by single motor species.

The specific design of these two motors appears to be optimized for their function in the mitotic spindle. HSET is distributed over the entire spindle (22, 36, 37), in agreement with a role for microtubule bundling, indicating that it may assist KIF11-driven sliding of microtubules toward the poles also in cells. This assisting role may explain why HSET levels contribute to controlling spindle length in a previously counterintuitive manner (37). Kinesin-14, however, also promotes pole focusing and bipolarization in cancer cells with too many centrosomes (38, 39) and in meiotic oocytes lacking centrosomes (40–42), supporting the notion that its function can differ in a spindle region where microtubule minus ends are enriched, cooperating there with the minus motor dynein.

Biological components with designs optimized for different tasks in living cells offer a variety of “tools” for integration into biomimetic materials, enriching the rulebook by which we understand macroscopic self-organized states, while giving rise to surprising new behaviors (14–16). Here, we have shown how motile cross-linkers determine the large-scale properties of an active material and how these properties can be controlled by varying not only cross-linker concentrations but also cross-linker properties. Despite our systematic exploration, we did not observe nematic and polar networks coexisting as in mitotic spindles. This most likely indicates that additional components are required to form spindle-like bipolar structures. For example, the local control of microtubule nucleation and dynamics may still need to be added in vitro for the active network to self-organize into spindle-like structures or other novel network patterns. This is an important challenge for the bottom-up assembly of active microtubule networks in the future.

## Methods

### I. Experiments.

#### Tubulin.

Tubulin was initially purified by two rounds of polymerization and depolymerization from porcine brain as described previously (43). *N*-ethylmaleimide (NEM; Sigma-Aldrich) tubulin was prepared from purified tubulin as described previously (44) without further recycling steps. To obtain small aliquots of highly active labeled and unlabeled tubulin, further recycling and labeling with CF640R-NHS (Sigma-Aldrich) or AlexaFluor568-NHS (Life Technologies) was performed as described previously (45). Final concentrations and labeling ratios were determined by nanodrop (extinction coefficient, 115,000 M^−1^ cm^−1^; molecular weight, 110 kDa) before adjustment, final ultracentrifugation, and aliquoting (unlabeled tubulin: 15-μL aliquots, 200 μM; labeled tubulin: 2-μL aliquots, 150 μM). Aliquoted tubulin was then snap-frozen and stored in liquid nitrogen.

#### Recombinant motor proteins.

Insect cell expression constructs for StrepTagII-mCherry-G_5_A-HSET (pJR291) and StrepTagII-KIF11-A_3_G_5_-monomeric green fluorescent protein (mGFP) (pJR303) have been described previously (6). To generate an N-terminally monomeric blue fluorescent protein (mBFP)-tagged HSET construct, similarly to previously described biotinylated constructs (46), HSET was cloned into a modified pFastBacDual vector (Thermo Fisher) containing a biotin-acceptor peptide (BAP), mBFP, and flexible linkers, resulting in the expressed construct BAP-G_5_-mBFP-ELG_6_A-HSET, which coexpresses with the biotin ligase BirA from separate promoters (pJR349). This construct was used for polarity-marked microtubule seed assays as an alternative to mCherry-HSET for use with the AlexaFluor568-labeled seeds—the biotin tag was unused and did not interfere with HSET activity.

Recombinant motor proteins were expressed in cultures of Sf21 insect cells (Thermo Fisher) according to the manufacturer’s protocols. Cell pellets from 500 mL of culture were snap-frozen and stored at −80 °C.

mCherry-HSET and KIF11-mGFP were purified by affinity chromatography using Strep-Tactin resin (Cytiva) as described previously (6). StrepTagII domains were cleaved by tobacco etch virus (TEV) protease after elution, and cleaved protein was further purified by gel filtration. KIF11-mGFP was supplemented with sucrose to 10% wt/vol before the final ultracentrifugation, and both proteins were snap-frozen in 5-μL aliquots at 1 mg/mL and stored in liquid nitrogen (*SI Appendix*, Fig. 7*A* and *B*).

Cell pellets expressing BAP-mBFP-HSET and BirA were resuspended in ice-cold lysis buffer (50 mM Na-phosphate, 300 mM KCl, 5mM MgCl_2_, 1 mM ethylene glycol bis(2-aminoethyl ether)tetraacetic acid (EGTA), 5 mM β-mercaptoethanol (β-ME), and 0.5 mM adenosine triphosphate (ATP), pH 7.5), supplemented with DNaseI (Sigma-Aldrich) and protease inhibitors (cOmplete EDTA-free, Roche). Lysate was homogenized in a glass douncer on ice, clarified by centrifugation at 50,000 rpm in a Ti70 rotor (Beckman Coulter) at 4 °C for 45 min, and filtered using disposable syringe filters (0.45-μm pore size, Millipore). Biotin from the media was removed by buffer exchange over HiPrep desalting columns (Cytiva) equilibrated in lysis buffer. The lysate was loaded onto immobilized monomeric avidin beads (Pierce/Thermo Fisher), and bound protein was washed with lysis buffer, HSET gel-filtration buffer (50 mM Na-phosphate, 300 mM KCl, 1 mM MgCl_2_, 1 mM EGTA, 5 mM β-mercaptoethanol, and 0.1 mM ATP, pH 7.5) supplemented with 5 mM ATP, and finally, HSET gel-filtration buffer. After eluting protein with HSET gel-filtration buffer supplemented with 5 mM biotin, biotin was again removed by running the eluate over a HiPrep desalting column equilibrated in HSET gel-filtration buffer. The protein was gel-filtered using a Superose 6 10/300 GL column (Cytiva), concentrated to 1 mg/mL, clarified in a table-top ultra-centrifuge, snap-frozen in 5-μL aliquots, and stored in liquid nitrogen (*SI Appendix*, Fig. 7*C*).

Final recombinant protein concentrations were determined by the Bradford assay (Bio-Rad) against a bovine serum albumin standard (Pierce/Thermo Fisher) on snap-frozen and thawed protein aliquots. Reported concentrations refer to monomers based on their predicted molecular weights.

#### Glass.

Polyethylene glycol (PEG)–passivated glass was prepared as described previously (45); however, glass for self-organization assays was prepared without biotin functionalization. Glass was generally used within 8 wk of preparation.

#### Microscopy stock materials.

Freshly prepared, ice-cold BRB80 buffer (80 mM K-PIPES, 1 mM MgCl_2_, and 1 mM EGTA, pH 6.8; prepared as a 5× stock and diluted to 1× before use) was used to resuspend β-casein (to 25 mg/mL, Sigma-Aldrich), glucose oxidase (to 40 mg/mL, Serva), and catalase (to 20 mg/mL, Sigma-Aldrich). Resuspended proteins were centrifuged at 80,000 rpm in a TLA 100 rotor (Beckman Coulter) at 4 °C for 15 min, aliquoted, snap-frozen in liquid nitrogen and stored at −80 °C. ATP and guanosine triphospate (GTP) were dissolved in MilliQ water to 100 mM each, adjusted to pH 7 using KOH, and filtered (0.22-μm pore size, Millipore) and aliquots were stored at −80 °C. Docetaxel (Sigma-Aldrich) was dissolved in dimethyl sulfoxide (DMSO) to 1 mM and filtered (0.22-μm pore size), and aliquots were stored at −80 °C. Glucose was dissolved in MilliQ water to 1 M, filtered (0.22-μm pore size), and stored at 4 °C.

#### Self-organization flow chambers.

On each day of experiments, flow chambers were assembled from the PEG-passivated glass using 80-μm-thick double-stick tape (Teva), generating a chamber about 5 mm wide and 7 mm long. Chambers for visualizing motion of polarity-marked seeds in self-organized networks were assembled using two layers of 10-μm-thick tape (Nitto Denko). With appropriately sized cover-glasses, multiple experiments could be performed in tandem. The assembled chambers were kept for up to one day at room temperature (RT) and placed on a 33 °C heat block just before preparing the assay.

#### Self-organization assay.

On each day of experiments, BRB80 was freshly diluted from 5× stock (stored for up to two weeks at 4 °C) and kept on ice. HSET and KIF11 gel-filtration buffer stocks without ATP or β-ME (stored at 4 °C) were supplemented with ATP or β-ME and kept on ice (HSET gel-filtration buffer: 50 mM Na-phosphate, 300 mM KCl, 1 mM MgCl_2_, 1 mM EGTA, 5 mM β-mercaptoethanol, and 0.1 mM ATP, pH 7.5; KIF11 gel-filtration buffer: 50 mM Na-phosphate, 300 mM KCl, 2 mM MgCl_2_, 10 mM β-mercaptoethanol, and 0.1 mM ATP, pH 7.5). Aliquots of catalase and glucose oxidase were mixed and diluted with BRB80 to make the oxygen scavenger mix containing 18.1 mg/mL catalase and 11.8 mg/mL glucose oxidase. The oxygen scavengers were centrifuged at 80,000 rpm in a TLA 100 rotor at 4 °C for 15 min, along with thawed β-casein to get rid of aggregates. The oxygen scavengers and β-casein were stored on ice.

Buffers and protein solutions were kept on ice, except for thawed docetaxel aliquots, which were kept at room temperature. Fresh KIF11 and HSET protein aliquots were thawed each experimental day, diluted appropriately in their respective gel-filtration buffers. Labeled and unlabeled tubulin aliquots were thawed before each experiment, mixed for a final labeling ratio of 3.5% CF640R, and diluted appropriately in BRB80. The final experimental solution contained 19.2 μL tubulin mix, 5.76 μL KIF11 dilution or KIF11 gel-filtration buffer, 5.76 μL HSET dilution or HSET gel-filtration buffer, 2.12 μL β-casein, and 2 μL oxygen scavenger mix, with 18.3 μL “docetaxel mix” to bring final buffer concentrations to 35 mM PIPES, 65 mM KCl, 1.68 mM MgCl_2_, 3.55 mM β-mercaptoethanol, 1 mM EGTA, 1 mM ATP, 0.64 mM GTP, 35 mM glucose, and 1 μM docetaxel (added freshly before each experiment from DMSO stock).

The sample was clarified by spinning at 13.3K rpm in a 4 °C table-top centrifuge for 5 min. The supernatant was transferred to a new tube on ice. The prewarmed flow chamber was washed, using blotting paper to draw the solution through the chamber, by flowing 50 μL of a “wash” solution with the same components as the final experiment solution, but KIF11, HSET, tubulin, or oxygen scavengers were replaced by their respective storage buffers. The wash solution was replaced by flowing through 50 μL of the sample solution. The chamber was sealed with silicone vacuum grease, then transferred to an inverted spinning-disk confocal microscope with an incubator at 33 °C. After locating the center of each chamber (between the tape and glass boundaries), imaging was initiated between 3 and 4 min after initial temperature shift. Exposure times for each laser line were 200 to 300 ms, with images taken at intervals of 1 min.

#### Polarity-marked microtubules.

Stable polarity-marked microtubules, with a bright minus end and a dim plus end, were prepared using AlexaFluor568 tubulin. Polarity labeling was performed as previously described (47). First, bright guanosine-5'-[(α,β)methyleno]triphosphate (GMPCPP) seeds were polymerized from 15 μM tubulin, with a total labeling ratio of 0.14, with 0.5 mM GMPCPP, diluted with BRB80 for 45 μL total volume, for 30 min at 37 °C. The seeds were spun down and washed twice, then resuspended with 45 μL BRB80 at RT. A polar extension mix (45 μL) containing 15 μM total of tubulin, of which 6 μM was N-ethylmaleimide (NEM)-tubulin, with AlexaFluor568 tubulin for a labeling ratio of 0.035, was mixed on ice with 1 mM GTP and 2 mM dithiothreitol (DTT). The mixture was transferred to 37 °C for 1 min before adding 10% of its volume of bright GMPCPP seeds and mixing gently, incubating for a further 20 min at 37 °C. The polymerized extensions were subsequently stabilized by the addition of docetaxel in two steps. After the 20-min polymerization, 5 μL BRB80 containing 2 mM DTT and 10 μM docetaxel was added. After 2 more minutes at 37 °C, the seeds were diluted with 150 μL BRB80 with 2 mM DTT and 10 μM docetaxel. To remove the excess unpolymerized tubulin, seeds were spun and resuspended three times over a 0.22-μm spin filter, in BRB80 with 2 mM DTT and 10 μM docetaxel, for a final volume of 50 μL. Seeds could be stored at RT for several hours before significant end-to-end annealing. Seeds could be further diluted in BRB80 with 2 mM DTT and 10 μM docetaxel and sandwiched between untreated coverslips to assess the efficiency of polarity marking. Of microtubules containing one bright segment, 63% had one dim segment (*n* = 747).

Microtubule gliding assays were performed as described previously (6). Flow chambers were prepared using untreated glass coverslips and 70 μm double-stick tape. The flow chambers were washed with 50 μL BRB80, followed by 50 μL of the assay “wash mix,” and incubated 2 min for β-casein to assemble on the surface. Then, 50 μL of 300 nM mGFP-KIF11 or avitag-mBFP-HSET in the self-organization assay buffer (without tubulin or oxygen scavengers) was added to the chamber and allowed to adhere to the surface for 5 min before washing out with two 50-μL washes of wash mix. Polarity-marked seeds were then diluted 1 in 200 in RT wash mix, and 50 μL of this polar seed sample was added to the chamber, which could then be sealed and examined on the confocal microscope. Motor attachment to the casein surface was confirmed by checking the fluorescence of the appropriate motor fluorophore. Of mobile microtubules with one bright and one dim segment, 100% were moved in the bright (minus) direction in KIF11 gliding assays (*n* = 35) and 97% in the dim (plus) direction in HSET gliding assays (*n* = 36). Additionally, microtubules with a repeating bright-dim segment pattern were also moved toward the bright end by KIF11, indicating that annealed polarity-marked seeds retained directionality.

When polarity-marked seeds were to be added to active microtubule networks, they were first diluted 1 in 10 in BRB80 (to 1 μM docetaxel). The experimental solution for polarity-marked seed embedded networks contained 14.2 μL tubulin mix, 5,76 μL KIF11 dilution, 5.76 μL HSET dilution or HSET gel-filtration buffer, 2.12 μL β-casein, 2 μL oxygen scavengers, and 18.3 μL docetaxel mix on ice. The seeds were further diluted 1 in 3 in RT BRB80 directly prior to mixing the final sample to avoid the addition of excess docetaxel while minimizing depolymerization. After spinning and before being added to the prewarmed, washed flow chamber, the solution was transferred into a RT tube, supplemented with 4.7 μL of the dilute polarity-marked seeds, and mixed gently before flowing into the chamber and imaging as described earlier.

#### Measurement of microtubule length distribution.

Chambers were prepared from one piece of biotin-PEG-passivated coverglass and one glass slide treated only with poly(L-lysine)-PEG (SuSoS), assembled with 80 µm double-stick tape (Teva). Microtubules were nucleated by docetaxel in a solution prepared as if for self-organization microscopy assays, without the inclusion of motor proteins. The sample tube was incubated at 33 °C using a heat block. Samples were taken at the indicated intervals and diluted 100 (for 15 µM tubulin) or 500 times (for 35 µM tubulin). For each sample, a rigor-mutant kinesin surface was prepared as described (46) by washing sequentially with NeutrAvidin (Thermo Fisher) and then a biotinylated, truncated *Drosophila melanogaster* kinesin-1 construct, with a point mutation that prevents ATP hydrolysis and promotes strong microtubule binding (Kin^rigor^). Then 50 µL of a sample was washed over the Kin^rigor^ surface; after washing out unbound microtubules with 50 µL of the assay buffer, the chamber was sealed with vacuum grease and imaged. Microtubules in a field of view were counted and measured by manually tracing them using Fiji (48).

### II. Simulations of Active Networks in Cytosim.

Simulations were performed with Cytosim (https://gitlab.com/f-nedelec/cytosim), an open source project. Cytosim solves the Langevin equation of motion of bendable microtubules with cross-linkers in viscous medium (34). Model parameters (see *SI Appendix*, Table S1) were chosen near the experimentally determined values or, when experimental data were missing, chosen to best match the network behavior observed experimentally in this study. Simulator and simulation configuration files are available on Zenodo, to allow reproducibility.

The system was simulated three-dimensionally with X and Y dimensions of 20 µm and a thickness in Z of 0.2 µm unless otherwise stated. The thickness of the box was chosen to minimize computational cost while still allowing multiple filaments to cross each other when overlapped. The box is reflective in Z and periodic in X and Y. When microtubule densities are stated in the text, they are reported as the number of microtubules in the quasi–two-dimensional box per X-Y area. Simulations were run with a time step of 0.005 s for a duration of 8,000 s (∼2 h), corresponding to experimental time scales.

#### Microtubule model.

Microtubules interact with each other via Hookean soft-core repulsive forces directed perpendicular to the filament axis so as to minimize sliding force parallel to the microtubule axis. Microtubules have discrete lattice binding sites covering 8 nm to which the cross-linker units can bind. Microtubules were nucleated from randomly distributed “seeds” at a rate such that most microtubules were nucleated at the very beginning of the simulation, similar to the experiment. Once nucleated, the plus end of the microtubule grows at a gradually decreasing speed, to mimic the limited availability of soluble tubulin pool as in the experiments (*SI Appendix*, Fig. 1). The time-dependent growth rate is expressed as $v_{g}(t)=\alpha \left[1-\left\{\sum^{\text{​}}L_{i}(t)\right\}/\text{Ω}\right]$, where α is the maximum growth speed, $\sum^{\text{​}}L_{i}(t)$ is the total length of all microtubules at time *t*, and Ω is the available amount of tubulin subunits in the system, a parameter expressed in micrometers.

To mimic the broadly distributed microtubule lengths in the experiment, we introduced variability in growth speed for each microtubule. We assigned a random speed α drawn from a normal distribution *N(0,1)* as α = $\alpha_{0}$ *|1 + 0.25 N(0,1)|*. The resulting speed distribution is centered near $\alpha_{0}$ and restricted to 0 to 2 µm/s. Fully grown microtubules did not undergo catastrophe, mimicking the docetaxel-stabilized microtubules in the experiment. At steady state, the average microtubule length is 2.5 µm, and thus shorter compared to the experiment. This decision was made as it was necessary to keep the simulation running time manageable on the computing cluster, in particular for large systems of which their dimensions exceed the individual microtubule lengths many times.

#### KIF11 model.

KIF11 was modeled as a symmetrical cross-linker with a pair of motor units connected by a Hookean spring with nonzero rest length. A freely diffusive motor in the medium can bind to a microtubule at a constant rate if it is within a specified binding distance. A bound motor can unbind with a force-dependent rate. Once bound, the motor walks stochastically on the one-dimensional lattice toward the plus end in a processive manner at a speed linearly dependent on the load. The velocity of a motor varies with force $\vec{f}$, as $v=v_{m}[1+\vec{f}\cdot \vec{d}/f_{s}]$, where $\vec{d}$ is the direction in which the motor would move along the filament if it was unloaded, *f_s_* is the stall force, and *v_m_* is the unloaded motor speed. Speed is thus decreased by antagonistic force as $\vec{f}\cdot \vec{d}<0$. KIF11 can cross-link a pair of microtubules with both units but cannot bind with both units to the same microtubule. The force is Hookean with $\vec{f}=k\left[1-e/|u|\right].\vec{u}$, for a spring of rest length e, stiffness k, and extension $\vec{u}$. The joints formed by the link rotate freely such that the angle between the cross-linked microtubules is not constrained.

As shown in previous simulations (6), microtubules organize into a nematic network when the steady-state motor profile along the microtubule is uniform. However, with short microtubules that stop growing and do not undergo catastrophe, KIF11 motors are able to reach and accumulate at the microtubule plus end, obstructing nematic network formation. This can be resolved by having longer microtubules in a larger simulation box, but this would incur a prohibitive computational cost. Instead, we set the motors to unbind immediately at the end of the microtubule, which allows nematic network organization with short microtubules and thus at reasonable computing time.

#### HSET model.

HSET was modeled as an asymmetric cross-linker with one motor unit and one diffusive unit. The motor is minus end directed and nonprocessive. When bound, the diffusive subunit steps stochastically to neighboring lattice sites on the microtubule. When the motor is not bound, the force is null and the diffusible subunit undergoes unbiased diffusion. However, when the motor is bound, the force would bias the plus and minus end–directed hopping rates in a thermodynamically consistent manner, as described previously (49, 50). Unlike KIF11 motor units, the HSET motor unit only binds transiently to a microtubule, taking on average two steps before unbinding. In comparison, the diffusive unit dwells for a relatively longer time on the microtubule. When reaching the end of a microtubule, both units can stay bound, allowing clustering of microtubule ends.

#### Quantification of microtubule motion.

To calculate the overall speed of microtubule motion in Figs. 5 and 6, we extracted the positions of microtubule minus ends at a time interval Δt. We then calculated the displacement component parallel to the microtubule axis. Averaging the displacement for all microtubules and dividing by Δt gave the overall averaged microtubule speed. A large Δt (640 or 1,280 s) was chosen such that the displacement due to diffusion is negligible relative to directed motion. The overall speed approaches the motor’s speed when all microtubules are cross-linked and slid continuously. In the absence of a motor, the overall averaged speed as extracted here has a small, nonzero value due to the rotational diffusion of the microtubules. As a matter of convention, a positive speed indicates minus-end leading unidirectional sliding (plus motor driven). Likewise, a negative sign indicates sliding with the plus-end leading.

#### Bundle and aster size calculation.

To calculate the size of bundles and asters as shown in Fig. 5*C*, we first identify clusters of microtubules cross-linked by the same type of cross-linker. The number of microtubules in the largest cluster is then reported as the bundle size and the aster size, respectively, for KIF11 and HSET. The size was normalized by the number of microtubules for convenience.

## Supplementary Material

Supplementary File

Supplementary File

Supplementary File

Supplementary File

Supplementary File

Supplementary File

Supplementary File

Supplementary File

Supplementary File

## Data Availability

The Cytosim simulation software are available at GitLab (https://gitlab.com/f-nedelec/cytosim) (51). All other study data are included in the article and/or supporting information.
