# Supplementary material for: Cross-linker design determines microtubule network organization by opposing motors
Source: Proc Natl Acad Sci U S A. 2022 Aug 12;119(33):e2206398119. doi: 10.1073/pnas.2206398119 (PMC9388136; doi:10.1073/pnas.2206398119)
Supplement: Supplementary File [file pnas.2206398119.sapp.pdf]

Supplementary information for

Crosslinker design determines microtubule network organization by opposing motors

Gil Henkin[1,2], Wei-Xiang Chew[1], François Nédélec[3,#], Thomas Surrey[1,2,4,#]

[1] Centre for Genomic Regulation (CRG), Barcelona Institute of Science and Technology (BIST), Dr Aiguader 88, 08003 Barcelona, Spain
[2] The Francis Crick Institute, 1 Midland Road, London, NW1 1AT, UK
[3] Sainsbury Laboratory, University of Cambridge, 47 Bateman Street, Cambridge, CB2 1LR, UK
[4] ICREA, Passeig de Lluis Companys 23, 08010 Barcelona, Spain

[#] Correspondence: thomas.surrey@crg.eu, fjn28@cam.ac.uk

This PDF file includes:
Figures S1 to S7
Table S1
Legend for movies

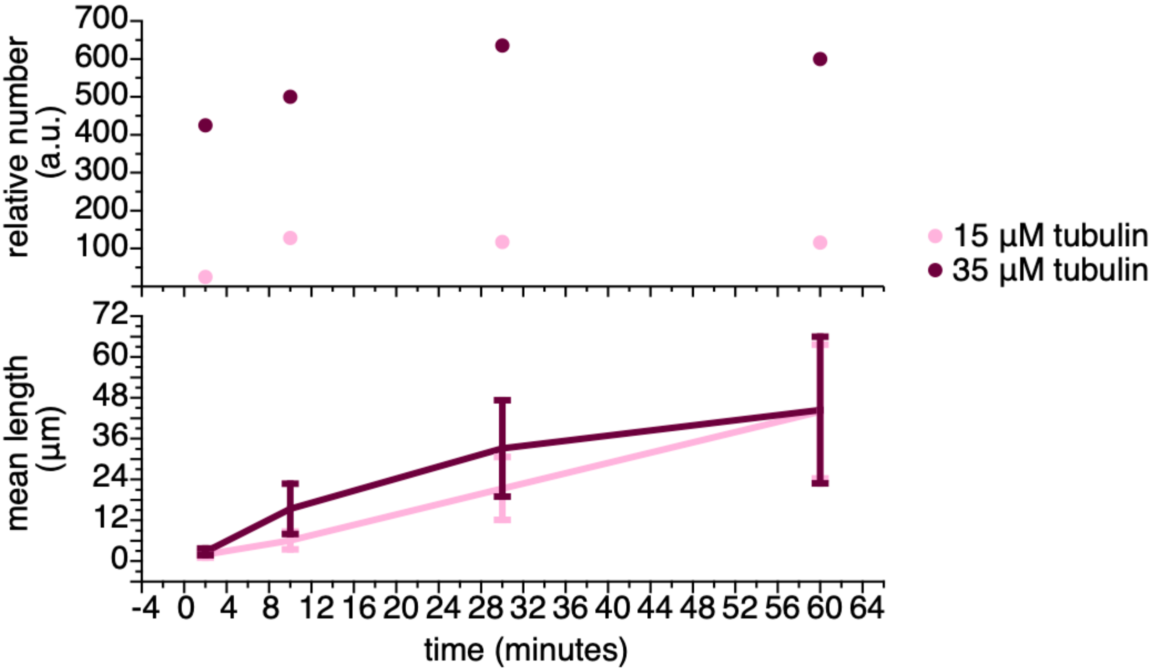

Suppl. Fig. 1 | Plots of relative number (above) and mean length (below) at different time points, taken from microscopy images of diluted samples immobilized on a rigor-mutant kinesin surface (surface prepared as published [1]. The samples for 35 µM tubulin were diluted five times more, so that the relative number reports five times the counted number in the field of view. Error bars on the mean length are standard deviations. For time points at 2, 10, 30, and 60 minutes the number of microtubules used to calculate the mean lengths were 25, 128, 117, and 116 (15 µM tubulin); 85, 100, 127, and 120 (35 µM tubulin).

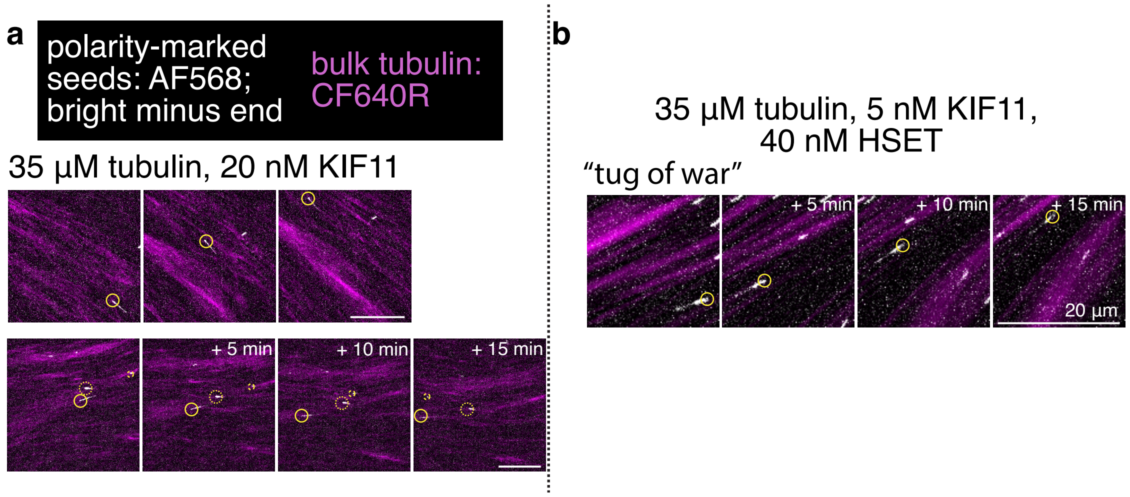

Suppl. Fig. 2 | Motility of polarity-marked microtubules with bright minus ends (white; AlexaFluor568) in high density microtubule networks (bulk tubulin, purple; CF640R). Yellow circles indicate microtubule minus end. **a**, 35 µM tubulin, 20 nM KIF11-mGFP: microtubules moving towards their minus ends. **b,** 35 µM tubulin, 40 nM mBFP-HSET, 5 nM KIF11-mGFP: microtubule undergoing saltatory, "tug of war" behavior.

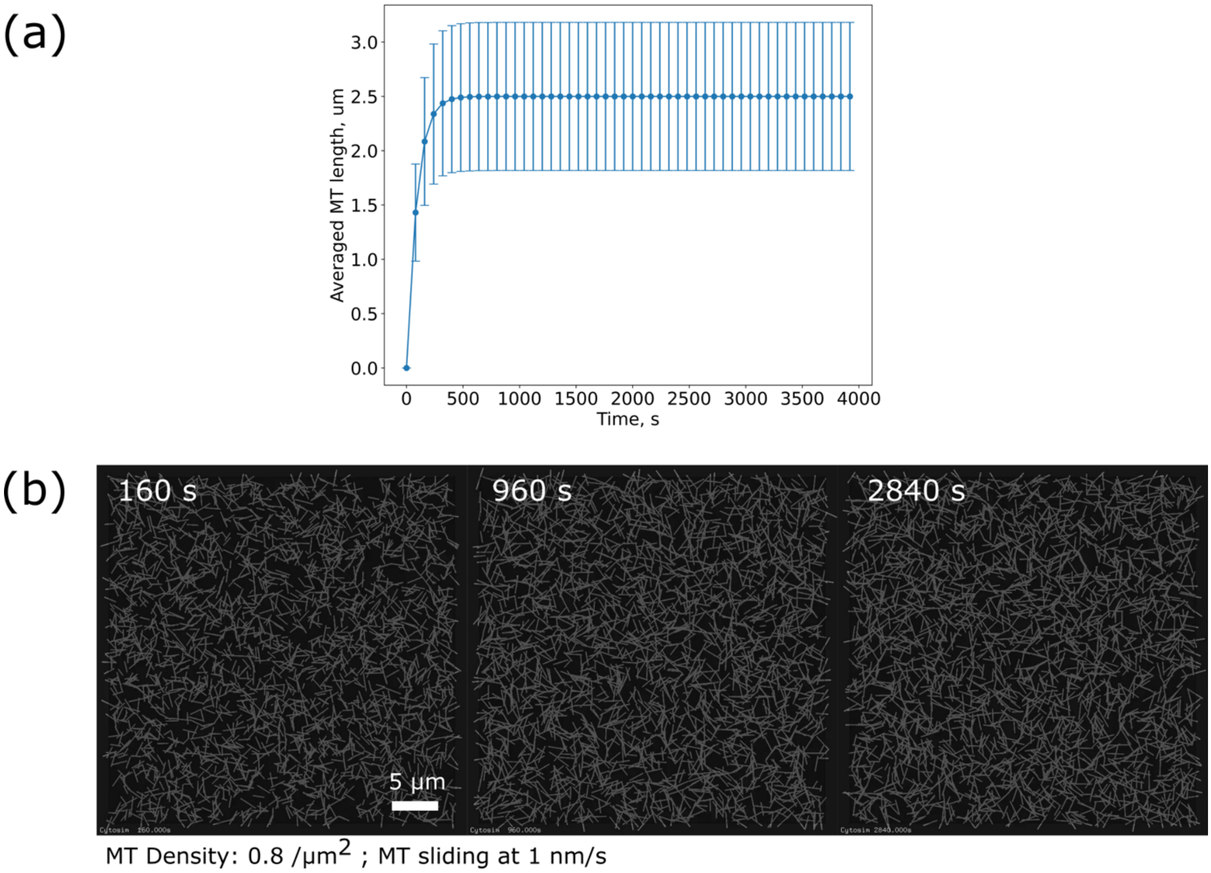

Suppl. Fig. 3 | Simulated microtubule nucleation and growth. a, Time series of the average length of 2,880 microtubules up to 4,000 s, from a simulation covering 60×60×0.2 μm. **b,** Corresponding time evolution of random microtubule organization in the absence of crosslinkers.

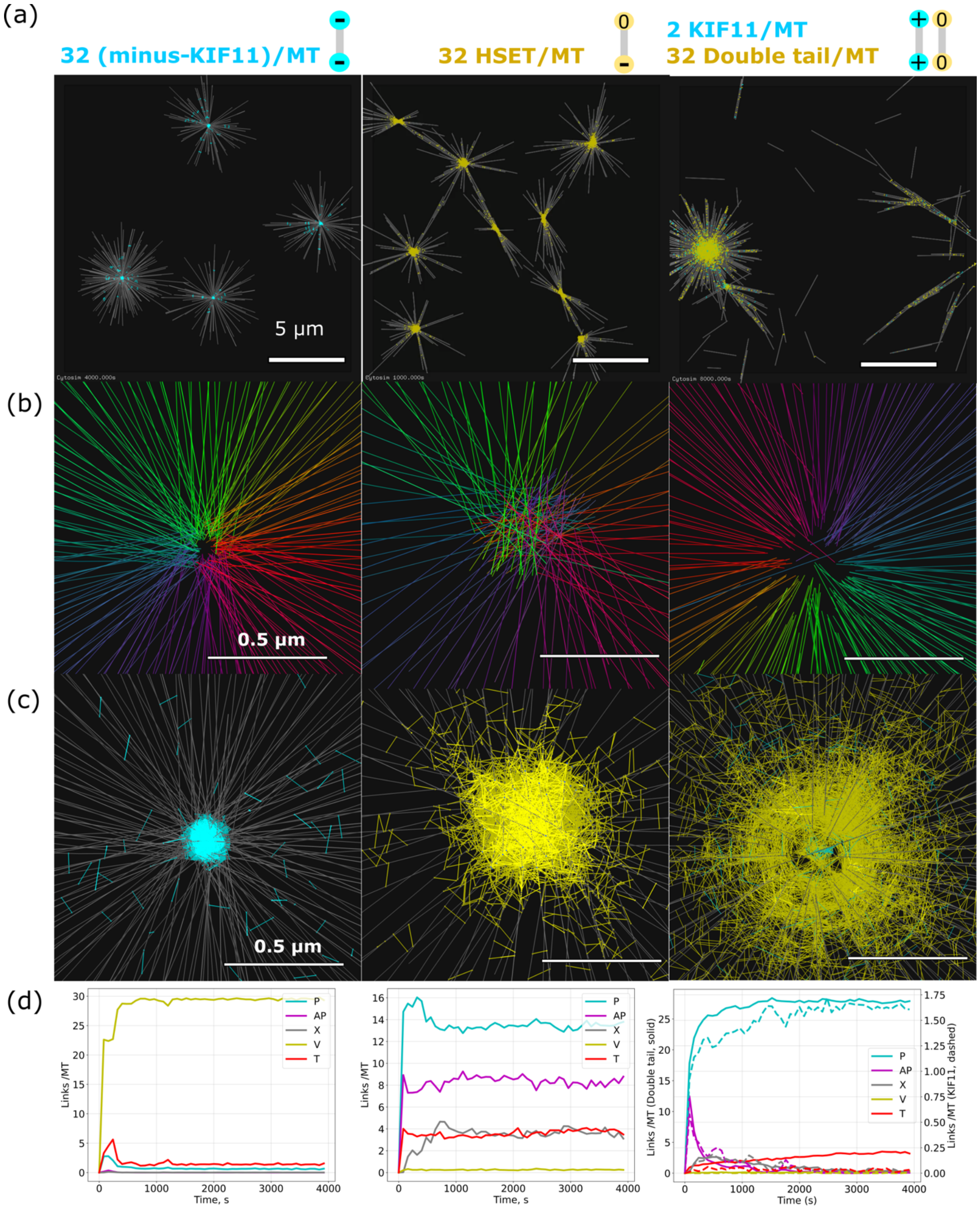

Suppl. Fig. 4 | Aster formed by different crosslinker types. a, Asters formed by KIF11 with hypothetical minus-end directed motors (left), by HSET (middle) and by plus-directed KIF11 mixed with double tail HSET variant (right). **b,** Close-up view of aster's center with microtubules colored according to its orientation. Microtubules' ends are focused (left), overlapped (middle) and focused (right) **c,** Localization of crosslinkers at the aster's center: minus KIF11 are narrowly localized (left), HSET are broadly localized (middle); double tails are localized with a radial gradient and KIF11 are narrowly localized at the center (right). **d,**

Time evolution of the connection types. Links made by crosslinkers were classified into 5 categories (V, T, P, AP, X) according to position of the crosslinker's unit with respect to the microtubules' ends, and the angle between the microtubules [2]. V-links connect microtubules' ends; T-links connect a microtubule's end to the side of another microtubule; P-links connect parallel microtubules where the internal angle is smaller than 60 degrees; AP-links connect antiparallel microtubules with an angle between 120 and 180 degrees; X-links connect microtubule' sides when these microtubules form an angle from 60 to 120 degrees. The count of links was normalized by the number of microtubules. Left: a high number of V-links indicate that the ends of microtubules are connected into focused asters; Middle: a prominence of T-links over V-links indicate that the ends of microtubules are not sharply focused. Instead, a lot of P, AP and X-links suggest that microtubules overlap and are bundled; Right: many P-links and few V-links by KIF11 (dashed line) indicate KIF11 bind mostly on parallel microtubules and fall off when reaching the plus ends. Both P and T links by double tail (solid lines) are high, indicating that the double tail is helping the aster to stay focused.

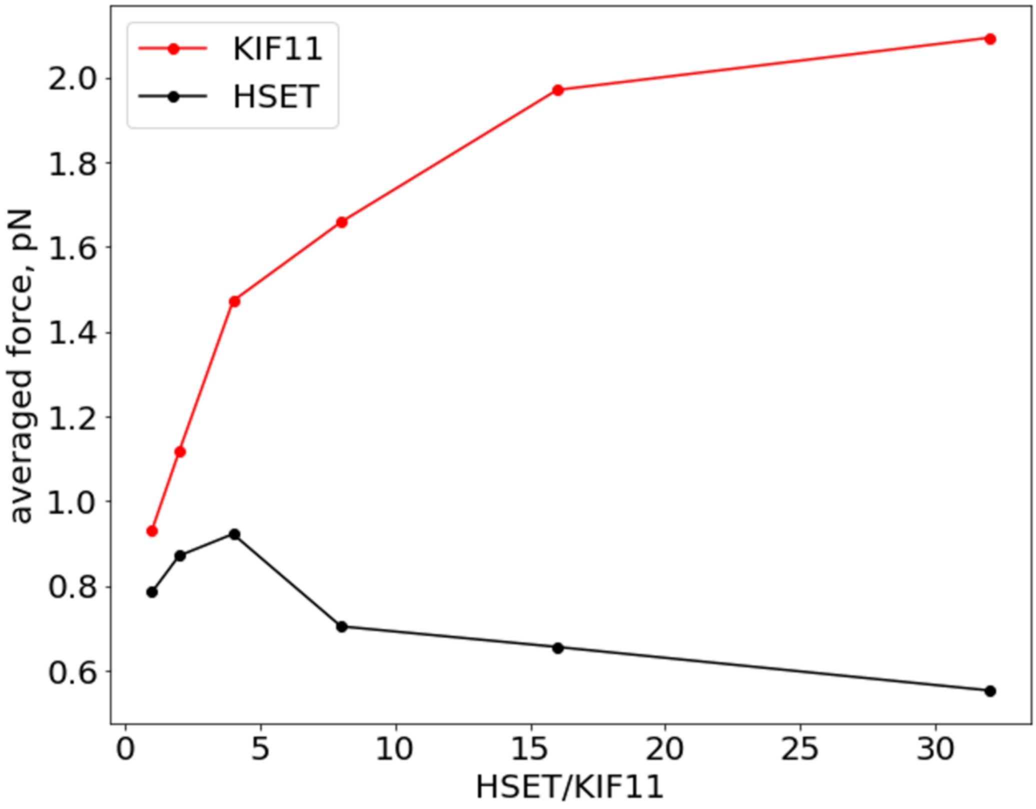

Suppl. Fig. 5 | Tension of crosslinked KIF11 and HSET. Averaged force exerted by KIF11 (red) and HSET (black) when sliding antiparallel microtubules. The force is plotted against the ratio of the number of HSET and KIF11. Overall, the tension produced is lower with HSET compared to KIF11, due to HSET's slippery tail. Tension of KIF11 increases when more HSET are introduced. Simulation contains 20 microtubules initialized in an antiparallel arrangement.

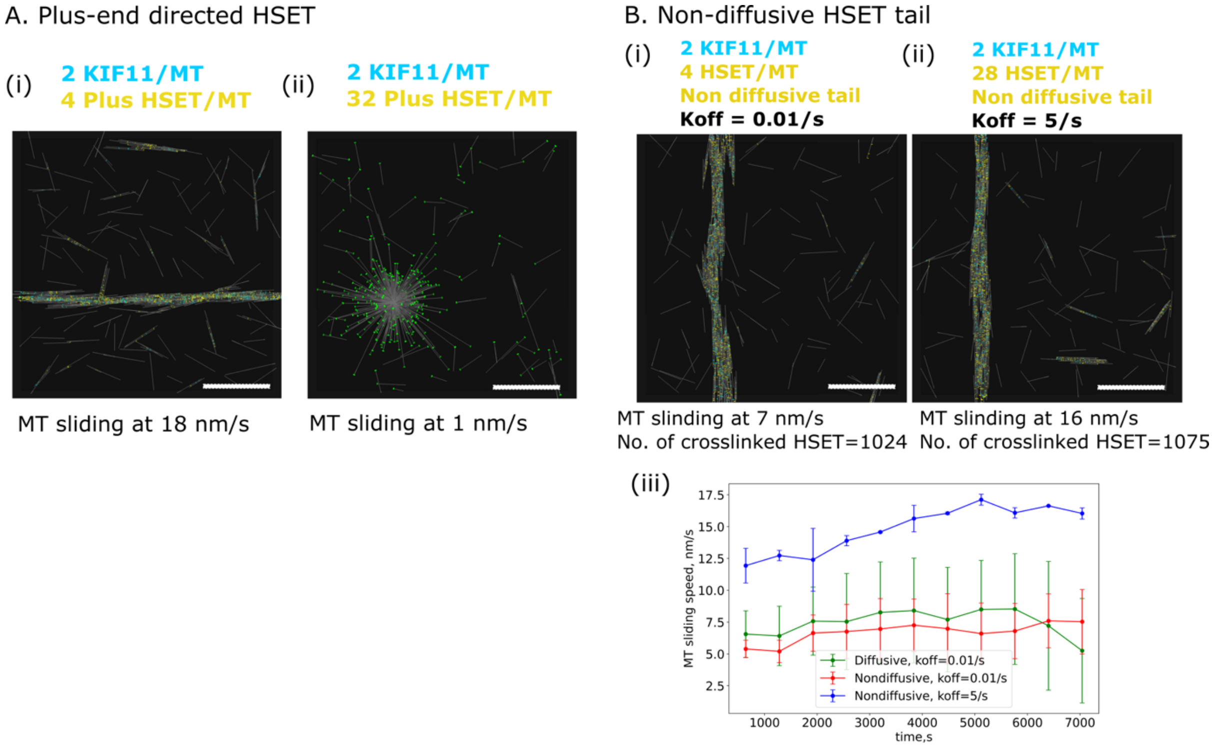

Suppl. Fig. 6 | A, Mixed motor simulation with plus end directed HSET. i, An extensile bundle forms when a small amount of plus end directed HSET (yellow) is mixed with KIF11 (cyan). ii, Inverted asters with the plus ends in the center form when a high amount of plus end directed HSET is mixed with KIF11. Minus ends of microtubules are highlighted in green. **B, Mixed motor simulation with HSET having a non-diffusive tail.** i, An extensile bundle forms when a small amount of HSET with a non-diffusive tail is mixed with KIF11. ii, When the unbinding rate of the non-diffusive tail is higher (5/s), increasing the amount of HSET to match the number of crosslinked motors at a given time (to 28 HSET/MT), an extensile bundle also forms. iii, Time series of microtubule motility in mixed motor networks for HSET having the natural diffusive tail (blue), a hypothetical non-diffusive tail with the same unbinding rate (red) and a non-diffusive tail with a higher unbinding rate (blue). Simulation space is 20×20×0.2 µm with 320 microtubules, covering 8,000 s in total. All simulations contain 0.8 microtubules /µm^2. Scale bar is 5 µm.

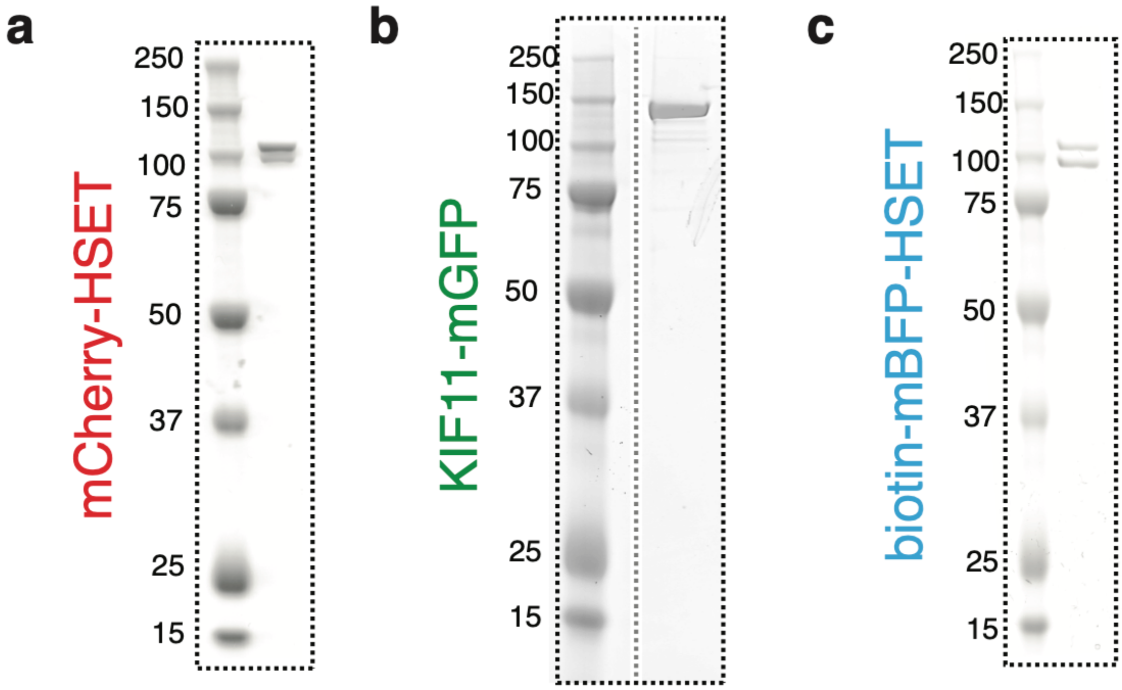

Suppl. Fig. 7 | Coomassie-stained SDS-PAGE gels of the three recombinant motor proteins. a mCherry-HSET, **b** KIF11-mGFP, **c** biotin-mBFP-HSET. Gel images are cropped and rotated to align to dashed boxes. KIF11-mGFP panel stitches protein marker and purified protein lanes from the same gel, translated on the x axis.

Table S1.

Parameter	Value	Note
**Simulation**		
Time step	5 ms	Small enough for convergence
Viscosity	0.02 pN s/μm^2	Close to water
Box thickness	0.2 μm	Thick enough for 3-4 filaments to cross
**Microtubule**		
Rigidity	30 pN μm^2	Ref. [3]
Steric radius	0.05 μm	Ref. [4]
Steric force constant	50 pN /μm	Constrained by thermal fluctuations and force of motors
Average length	2.5 μm	Optimum for computation
Growth rate	0.03 μm/s	Ref. [2].
Sensitivity of growth to force	1.67 pN	Ref. [3]
Nucleation rate	0.1 /s	Time for all microtubules to nucleate is short relative to total simulation time
**KIF11 motor**		
Stall force	5 pN	Ref. [5]
Unbinding force	5 pN	Refs. [6, 7]
Maximum motor speed	0.03 μm/s	Ref. [2]
Binding rate	0.5 /s	Estimated to match experiment
Unbinding rate	0.1 /s	From run length =0.3 μm as measured in Ref. [8]
Binding radius	0.16 μm	1.5x crosslinker rest length
**HSET motor**		
Stall force	5 pN	Same as KIF11
Unbinding force	5 pN	Same as KIF11
Maximum motor speed	- 0.08 μm/s	Within measured range in Refs. [2, 9, 10]
Binding rate	5 /s	Estimated to match experiment
Unbinding rate	5 /s	Same order of magnitude as in Ref. [11]
Binding radius	0.16 μm	1.5x crosslinker rest length
**HSET Diffusive tail**		
Diffusion constant	0.1 μm^2/s	Refs. [10, 11]
Binding rate	0.5 /s	Same as KIF11 motor
Unbinding rate	0.01 /s	Ref. [11]
Unbinding force	5 pN	Same as motor domain
Binding radius	0.16 μm	1.5x crosslinker rest length
**Motor complex**		
Rest length	0.105 μm	Microtubule radius (0.025 μm) + actual crosslinker rest length (0.08 μm)[12]
Link stiffness	100 pN/μm	Ref. [13]
**Discretization parameter**		
Filament segment length	0.5 μm	Optimum for computation
Filament lattice size	8 nm	Size of a tubulin dimer
Motor stepping size	8 nm	Same as lattice size

MOVIE LEGENDS

Movie 1 | 35 µM tubulin networks driven by **a,** 10 nM HSET and **b,** 60 nM KIF11. Related to Fig. 1.

Movie 2 | 10 µM tubulin networks driven by 20 nM HSET with **a,** 5 nM KIF11 and **b,** 20 nM KIF11. Related to Fig. 2.

Movie 3 | 35 µM tubulin networks driven by 5 nM KIF11 and 0 nM HSET, 5 nM HSET, 40 nM HSET and 100 nM HSET. Related to Fig. 4a.

Movie 4 | 15 µM tubulin networks driven by 5 nM KIF11 and 0 nM HSET, 5 nM HSET, 10 nM HSET and 40 nM HSET. Related to Fig. 4b.

Movie 5 | Polarity-marked seeds with bright minus ends translocating in a mixed-motor nematic network formed from 35 µM tubulin, 40 nM HSET, and 5 nM KIF11. Minus ends manually tracked and labeled with circles. **a, b,** Seeds sliding towards their minus ends in bundles. **c,** Seed exhibiting saltatory behavior. Related to Fig. 4d.

Movie 6 | Left, Movie of simulated KIF11-driven extensile nematic bundle in a thin 3D box (dimension: 60×60×0.2 µm). The 2,880 microtubules are colored in gray, and the 46,080 KIF11 in cyan. **Right,** Movie of simulated HSET-driven aster formation. Simulation space is 40×40×0.2 µm with 1,280 microtubules and 40,960 HSET. Related to Fig. 5.

Movie 7 | Left, Random microtubule organization in the presence of low amount of KIF11 (cyan); **Middle,** Extensile bundle formed when HSET (yellow) was added in the presence of KIF11 at 2:1 ratio. **Right,** Minus aster formation when HSET and KIF11 were mixed at 16:1 ratio. Simulation space is 60×60×0.2 µm with 2,880 Microtubules and 5,760 KIF11. Related to Fig. 6a.

Movie 8 | A portion of an extensile bundle formed in the presence of HSET and KIF11 at 2:1 ratio. **Left,** Microtubules (gray) with HSET (yellow) and KIF11 (cyan). **Right,** A few microtubules in the bundle are drawn as arrows (plus end = head), and colored randomly for this visualization. They move with the minus ends leading. Related to Fig. 6b.

SUPPLEMENTARY REFERENCES

1. Roostalu J, Cade NI, Surrey T. Complementary activities of TPX2 and chTOG constitute an efficient importin-regulated microtubule nucleation module. *Nat Cell Biol* 2015, **17**(11): 1422-1434.

2. Roostalu J, Rickman J, Thomas C, Nédélec F, Surrey T. Determinants of Polar versus Nematic Organization in Networks of Dynamic Microtubules and Mitotic Motors. *Cell* 2018, **175**(3): 796-808.e714.

3. Dogterom M, Yurke B. Measurement of the force-velocity relation for growing microtubules. *Science* 1997, **278**(5339): 856-860.

4. Nogales E, Whittaker M, Milligan RA, Downing KH. High-resolution model of the microtubule. *Cell* 1999, **96**(1): 79-88.

5. Meyhofer E, Howard J. The force generated by a single kinesin molecule against an elastic load. *Proc Natl Acad Sci U S A* 1995, **92**(2): 574-578.

6. Andreasson JO, Milic B, Chen GY, Guydosh NR, Hancock WO, Block SM. Examining kinesin processivity within a general gating framework. *Elife* 2015, **4**.

7. Carter NJ, Cross RA. Mechanics of the kinesin step. *Nature* 2005, **435**(7040): 308-312.

8. Kapitein LC, Peterman EJ, Kwok BH, Kim JH, Kapoor TM, Schmidt CF. The bipolar mitotic kinesin Eg5 moves on both microtubules that it crosslinks. *Nature* 2005, **435**(7038): 114-118.

9. Furuta K, Toyoshima YY. Minus-end-directed motor Ncd exhibits processive movement that is enhanced by microtubule bundling in vitro. *Curr Biol* 2008, **18**(2): 152-157.

10. Norris SR, Jung S, Singh P, Strothman CE, Erwin AL, Ohi MD*, et al.* Microtubule minus-end aster organization is driven by processive HSET-tubulin clusters. *Nat Commun* 2018, **9**(1): 2659.

11. Braun M, Lansky Z, Szuba A, Schwarz FW, Mitra A, Gao M*, et al.* Changes in microtubule overlap length regulate kinesin-14-driven microtubule sliding. *Nat Chem Biol* 2017, **13**(12): 1245-1252.

12. Kashina AS, Baskin RJ, Cole DG, Wedaman KP, Saxton WM, Scholey JM. A bipolar kinesin. *Nature* 1996, **379**(6562): 270-272.

13. Loughlin R, Heald R, Nedelec F. A computational model predicts Xenopus meiotic spindle organization. *J Cell Biol* 2010, **191**(7): 1239-1249.
